# Late-life obesity is a protective factor for prodromal Alzheimer’s disease: a longitudinal study

**DOI:** 10.18632/aging.102738

**Published:** 2020-01-25

**Authors:** Zhen Sun, Zuo-Teng Wang, Fu-Rong Sun, Xue-Ning Shen, Wei Xu, Ya-Hui Ma, Qiang Dong, Lan Tan, Jin-Tai Yu, Alzheimer’s Disease Neuroimaging Initiative

**Affiliations:** 1 Department of Neurology, Qingdao Municipal Hospital, Nanjing Medical University, Nanjing, China; 2 College of Medicine and Pharmaceutics, Ocean University of China, Qingdao, China; 3 Department of Neurology, Qingdao Municipal Hospital, Qingdao University, Qingdao, China; 4 Department of Neurology and Institute of Neurology, Huashan Hospital, Shanghai Medical College, Fudan University, Shanghai, China; 5 Data used in preparation of this article were obtained from the Alzheimer’s Disease Neuroimaging Initiative (ADNI) database (http://adni.loni.usc.edu). As such, the investigators within the ADNI contributed to the design and implementation of ADNI and/or provided data but did not participate in analysis or writing of this report. A complete listing of ADNI investigators can be found at: http://adni.loni.usc.edu/wp-content/uploads/how_to_apply/ADNI_Acknowledgement_List.pdf

**Keywords:** Alzheimer’s disease, body mass index, biomarker, brain volume, cognition

## Abstract

Higher body mass index (BMI) in late-life has recently been considered as a possible protective factor for Alzheimer's disease (AD), which yet remains conflicting. To test this hypothesis, we have evaluated the cross-sectional and longitudinal associations of BMI categories with CSF biomarkers, brain β-amyloid (Aβ) load, brain structure, and cognition and have assessed the effect of late-life BMI on AD risk in a large sample (n = 1,212) of non-demented elderly from the Alzheimer’s Disease Neuroimaging Initiative (ADNI) database. At baseline, higher late-life BMI categories were associated with higher levels of CSF Aβ42 (p=0.037), lower levels of CSF total-tau (t-tau, p=0.026) and CSF t-tau/Aβ42 (p=0.008), lower load of Aβ in the right hippocampus (p=0.030), as well as larger volumes of hippocampus (p<0.0001), entorhinal cortex (p=0.009) and middle temporal lobe (p=0.040). But no association was found with CSF phosphorylated-tau (p-tau) or CSF p-tau/Aβ42. Longitudinal studies showed that higher BMI individuals experienced a slower decline in cognitive function. In addition, Kaplan–Meier survival analysis revealed that higher late-life BMI had a reduced risk of progression to AD over time (p = 0.009). Higher BMI in late-life decreased the risk of AD, and this process may be driven by AD-related biomarkers.

## INTRODUCTION

Alzheimer’s disease (AD) is the most common form of dementia, affecting approximately 35.6 million people worldwide in 2010 and an estimated 115 million people by the year 2050 [1]. There are no current preventive or disease-modifying therapeutic measures. Therefore, the identification of modifiable risk factors is of major interest [2].

As an influential factor of AD, obesity plays different roles in AD during different life stages. Mid-life obesity is an established risk factor for AD dementia [3–5]. To be more precise, a high body mass index (BMI) in midlife is associated with an increased risk of developing diabetes, vascular pathology, hypertension and hypercholesterolemia that are all independent risk factors for cognitive impairment and AD dementia in later life [6, 7]. An influential meta-analysis concluded that being underweight, overweight or obese in midlife predicted higher risk of dementia, with much of the evidence resting on a single sample aged 40-45 at BMI assessment [8]. But the association between late-life BMI and AD is inconclusive. Multiple epidemiological studies have even illustrated late-life obesity as a protective factor for AD (the so-called obesity paradox) [9–11]. Higher late-life BMI is associated with decreased risk of dementia [12, 13], better cognition [14], slower progression of AD [15], and decreased mortality [16]. However, the evidence is not consistent, with some studies reporting an inverse association between obesity and AD risk [17] and others reporting no association [18, 19]. Inconsistencies might arise because previous studies have been quite small with short durations of follow-up.

More studies are warranted to elucidate and validate the relationship between late-life BMI and AD. The objectives of the current study were to examine the cross-sectional as well as the longitudinal association between late-life BMI and AD CSF biomarkers, imaging biomarkers and cognitive performance in 1,212 non-demented elderly individuals, using the Alzheimer’s disease neuroimaging initiative (ADNI) cohort. This study targeted older adults with normal cognition (NC) and mild cognitive impairment (MCI) excluded those with AD in order to determine whether a distinct relationship between late-life BMI and AD exists during the preclinical phase of AD.

## RESULTS

### Demographics

The demographics, clinical data, neuropsychometric test, and other characteristics of the study population are presented in Table 1. Of the 1,212 participants included in the study, 439 individuals were in the normal weight range; 518 were overweight; and 250 were obese. The overweight group had a higher proportion of females (*p* <0.001). The high BMI group had slightly less education (*p* = 0.022) and lower frequency of the ε4 allele of *APOE* gene (*p* = 0.001). In comparison, the prevalences of hypertension (*p* <0.001) and diabetes (*p =* 0.001) were higher in higher BMI categories, but there was no difference in the prevalences of hyperlipemia, cardiovascular diseases, and depression among the BMI categories.

**Table 1:** Demographic and clinical characteristics of participants according to their BMI.

**Participant features**	**Normal weight**		**Over weight**		**Obese**		*P* value*
Age (years)	439	76.2± 5.8	518	74.9± 5.5	250	74.1± 5.5	<0.001***
Female	439	218 (49.7%)	518	340 (65.6%)	250	125 (50%)	<0.001***
Education (years)	439	16.3± 2.8	518	15.9± 2.8	250	15.8± 2.9	0.022*
APOE Ɛ4 carrier status (0/1/2)	439	244/159/36	518	298/180/40	250	174/70/6	0.001**
NC:MCI	439	178:261	518	203:315	250	115:135	0.191
Coexisting diseases							
Hypertension	439	189 (43.1%)	518	254 (49.0%)	250	149 (59.6%)	<0.001***
Hyperlipemia	439	202 (46.0%)	518	255 (49.2%)	250	129 (51.6%)	0.340
DM2	439	21 (4.8%)	518	40 (7.7%)	250	31 (12.4%)	0.001**
Depression	439	83 (18.9%)	518	99 (19.1%)	250	52 (20.8%)	0.815
CVD	439	90 (20.5%)	518	121 (23.4%)	250	54 (21.6%)	0.561
CSF biomarkers (pg/ml)							
CSF Aβ42	307	172.2± 53.2	369	182.4± 55.3	184	189.5 ± 50.1	0.002**
CSF t-tau	306	88.4± 50.3	363	84.5± 52.4	182	69.9± 34.0	<0.001***
CSF p-tau	307	37.4 ± 21.0	369	37.7 ± 21.4	184	34.0± 20.3	0.126
CSF t-tau/Aβ42 ratio	306	0.60 ± 0.48	363	0.56± 0.49	182	0.42 ± 0.31	<0.001***
CSF p-tau/Aβ42 ratio	307	0.26 ± 0.20	369	0.25± 0.20	184	0.21± 0.18	0.021*
Structural MRI data (mm^3^)							
Hippocampus	380	6721± 1053	452	6999± 1055	222	7177± 1110	<0.001***
Entorhinal Cortex	373	3483± 763	454	3676± 705	214	3707± 735	<0.001***
Middle Temporal Lobe	373	19428± 2904	454	19875± 2805	214	20289± 2720	0.001**
Cognition (score)							
ADNI-MEM	439	0.41± 0.74	518	0.46± 0.76	250	0.61± 0.77	0.003**
ADNI-EF	439	0.34± 0.82	518	0.38± 0.88	250	0.34± 0.85	0.645
ADAS-Cog11	439	9.06± 4.51	518	8.72± 4.51	250	7.97± 4.54	0.009**
ADAS-Cog13	439	14.54± 7.00	518	13.89± 6.78	248	12.76± 7.10	0.006**
MMSE	439	28.1± 1.81	518	28.04± 1.75	250	28.36± 1.63	0.061

Data are given as n (%) or mean ± standard deviation

*P values for continuous variables are from one-way analysis of variance (ANOVA). *P* values for categorical data are from chi-square test.

*P-value <0.05;**P-value <0.01; ***P-value <0.001.

Abbreviations: BMI = body mass index; NC = Normal Cognition; MCI = Mild Cognitive Impairment; DM2 = Diabetes Mellitus Type 2; CSF =Cerebrospinal Fluid; Aβ42 = amyloid beta42; t-tau = total-tau; p-tau = phosphorylated-tau; CVD = Cardiovascular Diseases; ADAS-Cog = Alzheimer Disease Assessment Scale-cognitive subscale; MMSE = Mini-mental State Examination.

### BMI and CSF biomarkers

**Figure 1:**
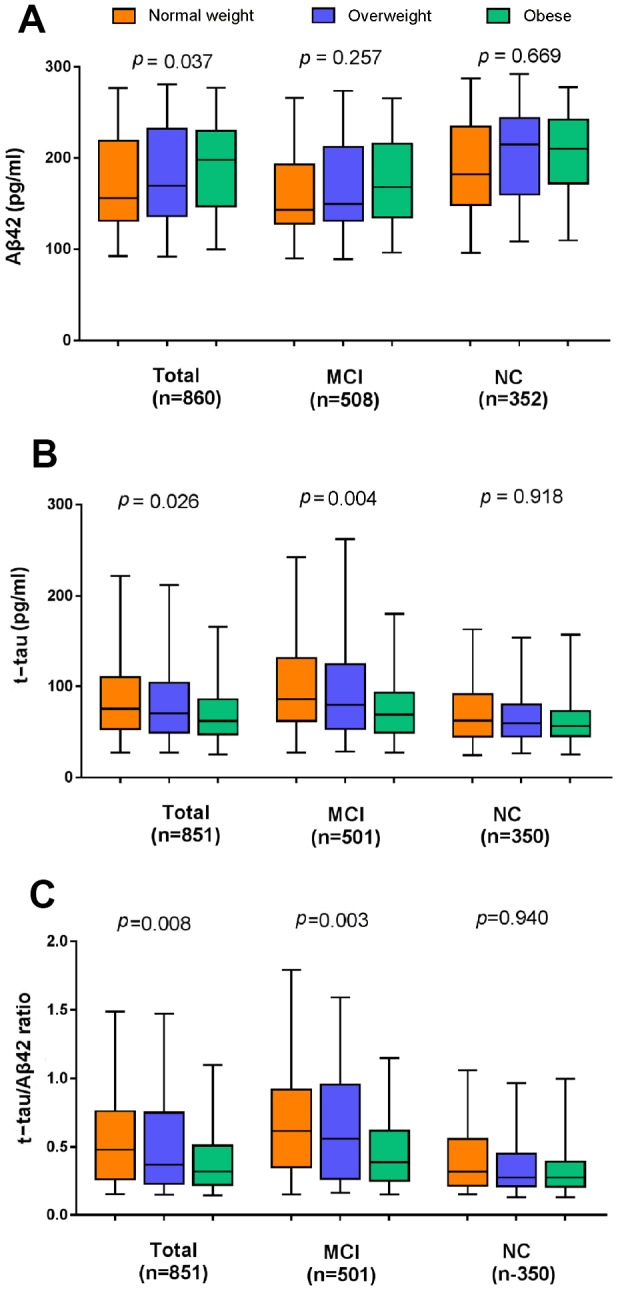
**The cross-sectional associations of BMI categories with CSF biomarker levels in the whole population and the subgroups stratified by cognitive diagnosis.** (**A**) Higher late-life BMI categories were associated with a higher level of CSF Aβ42 and lower levels of CSF t-tau (**B**) and CSF t-tau/Aβ42 (**C**). All analyses were adjusted for age, sex, education, cognitive diagnosis, ApoE ε4 status, CSF volume and prevalent comorbidities (i.e., hypertension, hyperlipemia, DM2, depression and CVD). Abbreviations: BMI = body mass index; MCI = mild cognitive impairment; NC = normal cognition; Aβ42 =β-amyloid42; t-tau = total-tau.

Regarding the whole population, BMI was positively correlated with the levels of CSF Aβ42 (Spearman r = 0.157), and inversely correlated with the levels of CSF t-tau, CSF p-tau, CSF t-tau/Aβ42 and CSF p-tau/Aβ42 (r = -0.152, -0.069, -0.178 and -0.119, respectively). Multiple linear regression models confirmed the Spearman’s associations of BMI categories with CSF Aβ42 (β = 0.335; p = 0.037), CSF t-tau (β =-0.023; p = 0.026) and CSF t-tau/Aβ42 (β = -0.093; p=0.008) after controlling for age, sex, education, cognitive diagnosis, ApoE ε4 status, extracted CSF volume and comorbidities (Figure 1) in the total study population. But CSF p-tau levels (p = 0.550) and CSF p-tau/Aβ42 (p = 0.180) were not significantly correlated with BMI in this model. When stratified by cognitive diagnosis, higher BMI categories were associated with lower levels of CSF t-tau (β= -0.093; p=0.004) and CSF t-tau/Aβ42 (β= -0.123; p=0.003) only among those with MCI, but were not associated with CSF Aβ42 or p-tau in any of the cognitive diagnostic groups (Figure 1).

When adjusted for age, sex, education, cognitive diagnosis, ApoE ε4 status and comorbidities, we found no statistically significant longitudinal association between baseline BMI categories and the change rates of CSF markers during a 7-year follow-up in either the whole population or in the two subgroups (MCI and NC).

### BMI and Aβ PET imaging

We looked at the correlations between BMI and regional Aβ burden in different bilateral regions (hippocampus, entorhinal cortex and middle temporal) and did not detect any statistically significant correlations between them in the whole sample. After adjusting for age, sex, education, cognitive diagnosis, ApoE ε4 status and comorbidities, higher baseline BMI categories was associated with lower load of Aβ in the right hippocampus in the total population (β = -0.010, p=0.030) and NC subjects(β = -0.011, p=0.039), but not MCI subjects. However, no associations were observed in other regions of interest (ROIs) between BMI categories and regional Aβ load.

Moreover, the longitudinal analyses showed that BMI categories at baseline were not significantly associated with the change rates of cerebral Aβ deposits during a 2-year follow-up in the overall sample nor in the subgroups, after adjusting for all variables mentioned above.

### BMI and brain structures on MRI

**Figure 2:**
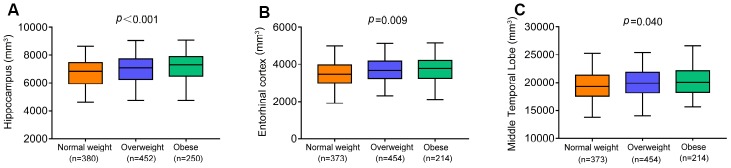
**The cross-sectional associations of BMI categories with brain volumes in the whole population.** Higher BMI categories were associated with larger volumes of hippocampus (**A**), entorhinal cortex (**B**) and middle temporal lobe (**C**). All analyses were adjusted for adjusting for age, sex, education, cognitive diagnosis, ApoE ε4 status, prevalent comorbidities (i.e., hypertension, hyperlipemia, DM2, depression and CVD) and total intracranial volumes. Abbreviations: BMI = body mass index;CVD = Cardiovascular Diseases;DM2 = Diabetes Mellitus Type 2.

We examined the correlations of BMI with the volumes of hippocampus, entorhinal cortex, and middle temporal lobe in the total non-demented elders and significant positive correlations were observed in the total sample (hippocampus, r = 0.200; entorhinal cortex, r = 0.149; middle temporal lobe, r = 0.142). Moreover, linear regression analyses controlling for demographic characteristics, comorbidities and total intracranial volumes (ICVs) revealed that higher BMI categories were associated with larger volumes of hippocampus (β = 129.579, p<0.001), entorhinal cortex (β =74.083, p=0.009) and middle temporal lobe (β =204.24, p=0.040; Figure 2) in the whole population. ICVs in regression models were standardized to z scores to facilitate comparisons. We also tested whether the influence of BMI categories differed between diagnostic groups at baseline. Higher BMI categories were associated with larger volumes of entorhinal cortex (β=92.612, p=0.013) in NCs but not in those with MCI. However, higher BMI categories were associated with larger volumes of hippocampus (β=169.954, p=0.002) and middle temporal lobe (β =277.260, p=0.048) in those with MCI but not in NCs.

Longitudinal studies found no statistically significant differences in the associations between BMI categories and the change rates of 3 ROIs over the 10-year follow- up in linear mixed-effects models adjusting for the same confounders in the whole population, in the NC or MCI subgroups.

### BMI and cognitive measures

We found significant correlations of BMI with ADNI-MEM score, ADAS-Cog11, ADAS-Cog13 and MMSE, but not with ADNI-EF in the whole population (ADNI-MEM, r = 0.094; ADAS-Cog11, r = -0.010; ADAS-Cog13, r = -0.106; MMSE, r = 0.189). Cross-sectionally at baseline, higher BMI categories were associated with better ADNI-MEM score (β = 0.060, p = 0.008), ADAS-Cog11 (β = -0.092, p = 0.0107) and ADAS-Cog13 (β = -0.143, p = 0.017), but were not associated with other cognitive measures in the entire sample (MMSE, p = 0.579; ADNI-EF, p = 0.370), after adjusting for demographic characteristics and prevalent comorbidities. When stratified by cognitive diagnosis, there was no association between BMI and cognitive performance in both control and MCI groups.

**Figure 3:**
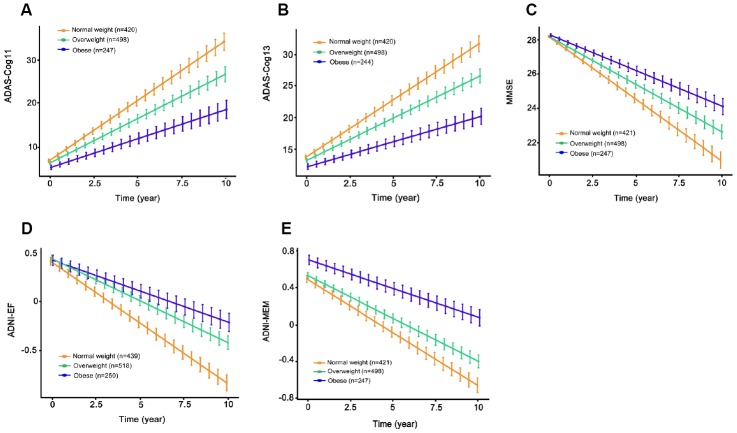
**The longitudinal associations of BMI categories with cognitive performance in the entire sample.** Higher BMI categories were associated with slower rates of cognitive decline on ADAS-Cog11 (**A**), ADAS-Cog13 (**B**), MMSE (**C**), ADNI-EF (**D**) and ADNI-MEM (**E**) over the following ten years. All analyses were adjusted for age, sex, education, cognitive diagnosis, ApoE ε4 status, as well as prevalent comorbidities (i.e., hypertension, hyperlipemia, DM2, depression and CVD). Abbreviations: BMI = body mass index; ADAS-Cog = Alzheimer Disease Assessment Scale-cognitive subscale; MMSE = Mini-Mental State Examination.

Longitudinally, higher baseline BMI categories were associated with slower rates of cognitive decline by all measures in the whole population (ADAS-Cog11, β = -0.351, p=7.52e-05; ADAS-Cog13, β = -0.491, p = 3.47e-06; MMSE, β = 1.56e-01, p = 0.001; ADNI-EF, β = 3.15e-02, p = 2.87e-05; ADNI-MEM, β = 2.60e-02, p = 4.07e-05; Figure 3) and in the MCI group (ADAS-Cog11, β = -0.535, p <0.001; ADAS-Cog13, β = -0.751, p = 5.64e-06; MMSE, β = 0.222, p = 0.002; ADNI-EF, β = 0.046, p = 2.87e-05; ADNI-MEM, β = 0.036, p < 0.001) over the following ten years. By contrast, in the NC group, there were no statistically significant differences in the association with rates of cognitive decline among the three BMI categories.

### BMI and AD progression

**Figure 4:**
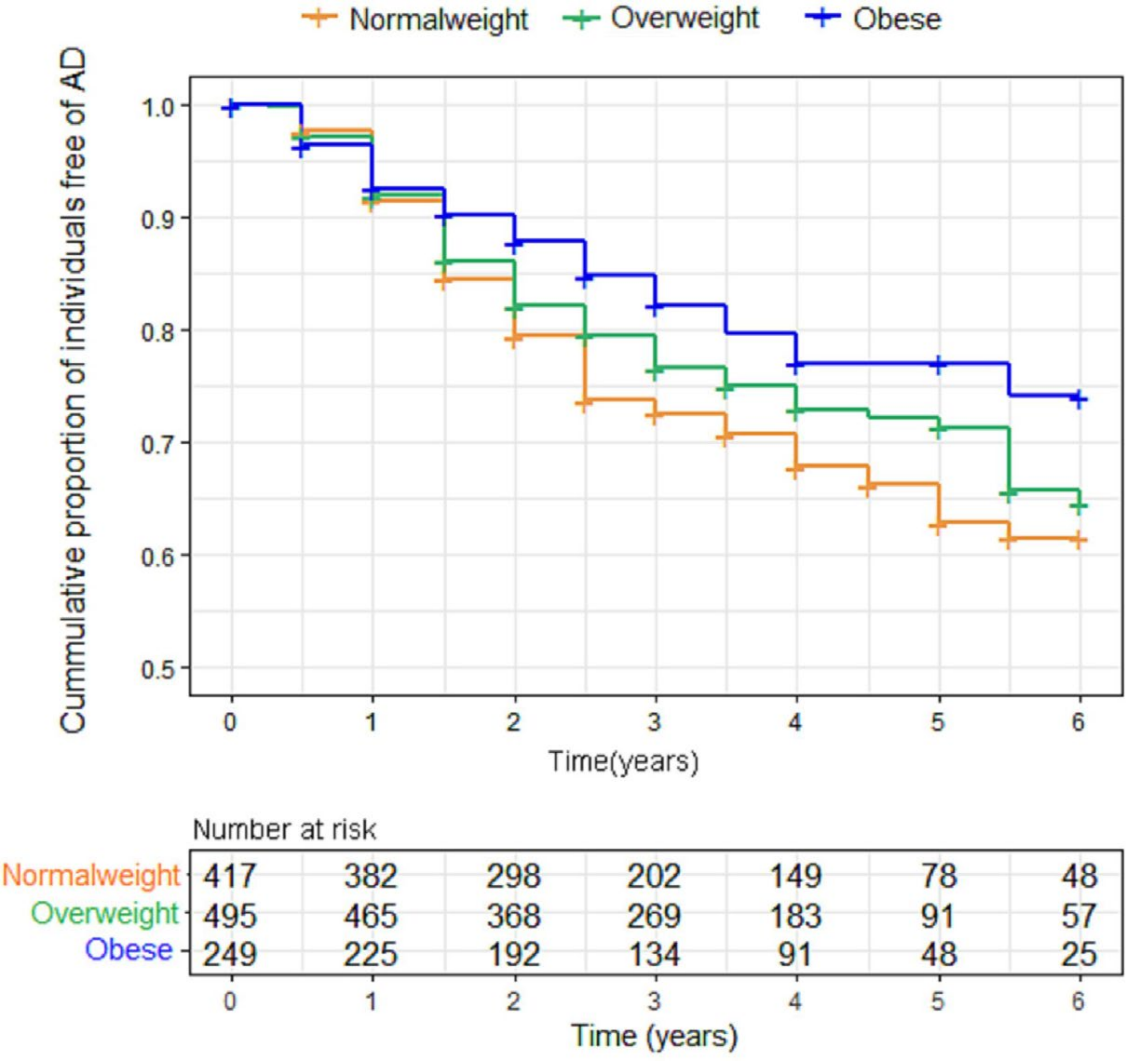
**Kaplan-Meier curves comparing survival free from progression to AD according to baseline body mass index (BMI) categories.** Individuals with higher BMI categories had a lower risk of progression to AD over the following six years. Abbreviations: BMI = body mass index.

When we examined the effect of BMI on AD risk using the Kaplan–Meier test, we found that BMI was inversely associated with AD risk. Indeed, the obese elderly had a lower risk of progression to AD over the following six years, compared to overweight and normal weight individuals (p = 0.009). However, further adjustment for confounding variables made little difference to this inverse association between BMI and AD risk (p=0.160). Figure 4 shows the Kaplan-Meier curves for the baseline BMI groups.

## DISCUSSION

In this study, we examined the influence of BMI on AD CSF and imaging biomarkers, brain Aβ load, as well as cognitive performance in a large multicenter cohort (ADNI) of non-demented older individuals. Our findings clearly indicated that higher baseline BMI was associated with higher levels of CSF Aβ42, lower t-tau and t-tau/Aβ42, lower load of Aβ, as well as larger brain volumes of AD-vulnerable regions. Further, the longitudinal data suggested that individuals with higher late-life BMI had less cognitive decline. Finally, Kaplan–Meier survival analysis revealed that higher late-life BMI had a lower incidence of conversion to AD. These analyses support the obesity paradox that has been observed across different studies where higher BMI in late life decreases AD risk [20].

Consistent with previous studies, our study has demonstrated that in non-demented subjects there were associations between late-life BMI and core feasible CSF biomarkers (Aβ42 and t-tau) which have superior accuracy for the detection of AD and could indirectly assess the extent of AD pathology in the brain [19, 21, 22]. But our study found not all the associations were statistically significant in different diagnostic subgroups using the ADNI database. Our results showed that baseline BMI categories were associated with CSF t-tau but not with CSF Aβ42 in the brains of MCI individuals, suggesting that individuals with MCI who are normal weight were more likely to have tau-induced cognitive impairment compared to those who are overweight or obese. However, in NC group, there was no association between BMI and the level of CSF tau. The above findings were consistent with those of Mathys et al.’s study showing that BMI was significantly associated with the level of tau in the MCI group, but not in the NC group [21]. We also observed that higher BMI categories were associated with a higher level of CSF Aβ42 in the total participants, but not in the MCI group or the NC group, which contradicted prior smaller studies showing an association in the MCI group [21, 22]. The potential explanations for this inconsistency may be the different numbers of MCI individuals for association analysis and whether BMI was assessed as a categorical variable or a continuous variable.

It has been suggested that many years prior to the appearance of clinical signs of AD, the deposition of Aβ occurs within the neocortex and hippocampus [23]. Interestingly, the results of our cross-sectional tests indicate that through serial assessment of Aβ using florbetapir PET imaging, subtle but significant lower Aβ load are present in brain region of right hippocampus among subjects with higher BMI, and that the protective effect of late-life obesity against AD dementia may be mediated by reducing Aβ accumulation in the hippocampus. However, BMI categories were not associated with the longitudinal rates of Aβ accumulation in the follow-up study of 2 years. This result may have been influenced by the short follow-up time, which may be too abbreviated to verify the longitudinal association between late-life BMI and Aβ accumulation.

As for MRI measures, our study demonstrated that higher late-life BMI categories were associated with larger volumes of hippocampus, entorhinal cortex and middle temporal lobe at baseline. This association may, at least partly, underlie the link between high BMI at late-life and a decreased risk of AD, compared to overweight/normal weight individuals. In our study, BMI-related atrophy was observed in the medial temporal lobes, including the entorhinal cortex and hippocampal subfields, which have been extensively studied as two brain regions first affected by AD. Both brain regions are involved in modulating appetite, olfaction and taste, as well as weight regulation [11, 24–26]. Recent research suggested the hippocampus has direct connections to hypothalamic nuclei and other brain networks, which underlies its role in regulation of food intake and body weight [27]. Although we did not directly assess the mechanisms underlying the link between BMI and neurodegenerative processes which could lead to brain atrophy, this relationship could be mediated partly by Aβ42 and tau. This was consistent with the previous findings about the involvement of BMI in the pathogenesis of AD [28–30].

Longitudinal studies have the advantage of following cohorts of individuals over prolonged periods of time, making themselves an ideal approach to observing disease development and identifying patterns, correlates and possible causes of changes that occur with age [31]. The analysis using longitudinal follow-up data available from our study participants showed that although cognitive function decreased over the follow-up period, obese individuals experienced a slower decline in cognitive function, compared with normal weight and overweight individuals. This finding is in line with recent studies showing that obesity was associated with a lower risk of cognitive decline in an elderly population, suggesting the protective association of high BMI with cognitive performance [32, 33]. The possible mechanism accounting for the protective association between obesity and cognition was explained by the hormone leptin which is mainly secreted by the adipose tissue [34]. An increased level of leptin regulates synaptic plasticity in the hippocampus, hence decreasing AD. Other mechanisms, including involving neurodegenerative and cognitive reserve, need more exploration. In the present study, however, no significant association was found between higher BMI categories and longitudinal cognitive changes in the subgroup with NC. A possible explanation for this finding could be that protective effect of high BMI on cognitive performance became increasingly obvious with disease progression.

Finally, we found that a higher late-life BMI at baseline was associated with a reduced risk of progression to AD. After adjustment for age, sex, education, cognitive diagnosis, ApoE ε4 status and comorbidities, this effect became nonsignificant among an elderly population. However, in a population of more than three hundred thousand olderly individuals, Qizilbash and colleagues found a lower risk of dementia in people who are overweight and obese, after adjustment for nine potential confounders: age, sex, smoking, alcohol, history of myocardial infraction, stroke and diabetes, use of recent anti-hypertensive drugs, and statins [35]. In other studies adjusted for several important potential confounders, researchers also reported that obesity at baseline predicted a lower risk of AD in elderly people [20, 36, 37]. Additionally, the results from our study confirmed a higher late-life BMI was associated with lower AD-related biological biomarkers in prodromal AD, larger brain volumes of AD-vulnerable regions and better cognitive performance. This finding implies a potentially protective effect of obesity on AD in the elderly. Therefore, it may be an inaccurate interpretation of the nonsignificant association of BMI and AD risk, and further studies with larger sample size are needed to assess the impact of BMI on AD risk.

The present study has several limitations. First, with the increase in adiposity, the precision of BMI measurement in older adults can be affected by age-related loss of lean body mass [38, 39]. Hence, future studies should examine other body composition metrics, such as waste circumference and waist-to-hip ratio. Second, there was a small number of participants with underweight, which limited the conclusions we could make about clinical progression among these individuals. Third, although longitudinal studies can potentially characterize the associations of BMI with change rates of CSF AD biomarkers and cognitive decline, they can be confounded by participant dropout rates. There was some loss to follow-up in our study and we didn’t correct for attrition bias. Fourth, we could not exclude the possibility of reverse causality in the association between BMI and AD. Fifth, except the three brain regions, others, such as parahippocampus, amygdala, precuneus cortex, and posterior cingulate, are also found to be associated with AD [40]. Despite the limitations, this study has several strengths, including a relatively large sample, longitudinal design, standardized clinical diagnosis of dementia, and several important adjusted confounders.

Overall, higher baseline late-life BMI was associated with lower levels of AD biomarkers, lower Aβ load, higher brain volumes, slower cognitive decline. These associations suggest that the obese elderly are at a decreased risk of AD. Future studies are warranted to better elucidate the mechanism underlying these associations.

## MATERIALS AND METHODS

### ADNI

**Figure 5:**
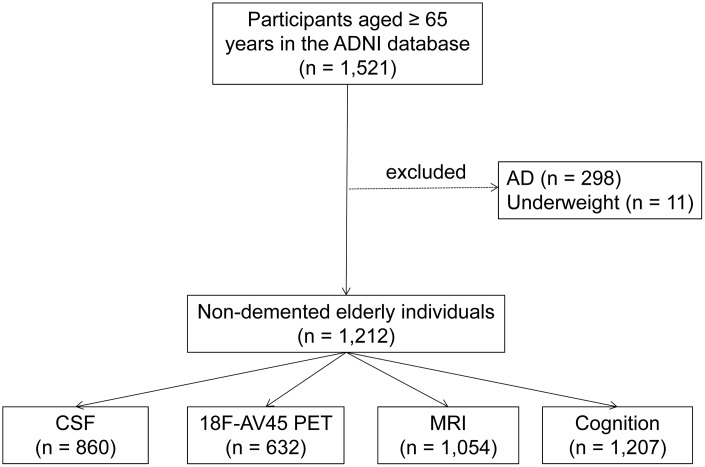
**Flowchart showing the samples used in this work and the subsets utilized for analyses.** Abbreviations: AD = Alzheimer's disease; CSF =Cerebrospinal Fluid.

Data used in the preparation for this article were obtained from the ADNI database (http://adni.loni.usc.edu). As an ongoing project, the ADNI was launched in 2003, sponsored by many co-investigators from a range of academic institutions and private corporations. The goal of ADNI is to determine biological markers of Alzheimer’s disease through neuroimaging, genetics, and neuropsychological tests as well as other measures to develop new treatments, monitor their effectiveness, and lessen the time of clinical trials. The ADNI has recruited more than 1800 adult participants (aged 55-90 years) with NC, MCI, or mild AD from over 50 sites across the United States and Canada in three phases (1, GO, and 2). The present study consisted of 1,223 non-demented elders which were ≥65 years at the time of the baseline scan. Because few subjects were in the underweight group (BMI<18.5 kg/m^2^, n = 11), we excluded them at baseline to avoid unmeasured confounding associated with baseline severe disease leading to very low BMI. Finally, 1,212 individuals were included in our study, including 714 MCI and 498 NC (Figure 5). The study was approved by the institutional review boards of all participating centers and all participants provided informed written consent.

### CSF collection and quality control

The ADNI Biomarker Core has previously described the procedures for acquiring and processing CSF for biomarker analysis. Briefly, CSF samples from each participant were drawn in the morning after an overnight fast and were collected into collection tubes. The CSF was transferred to polypropylene tubes and was immediately cooled to 4°C on dry ice until processing in the ADNI Biomarker Core laboratory at the University of Pennsylvania Medical Center, which occurred within 24 hours after collection. Aliquots (0.5 ml) were prepared from these samples after 1 h of thawing at room temperature and stored in polypropylene tubes at –80°C. INNO-BIA AlzBio3 (Innogenetics, Ghent, Belgium; reagents for research use only) immunoassay kit–based reagents were used to measure Aβ42, total-tau (t-tau), and phosphorylated-tau (p-tau).

### 18F florbetapir AV45 PET imaging and analysis

18F florbetapir-PET was used to estimate cerebral Aβ load. Data for 18F-Florbetapir imaging was acquired from UC Berkeley-AV45 analysis database (see http://adni.loni.usc.edu/data-samples/access-data/), in which three bilateral regions (hippocampus, entorhinal cortex and middle temporal) were selected as ROIs to study. In brief, summed images 50–70 minutes postinjection were coregistered to each participant’s MRI to enable alignment of FreeSurfer ROIs. Meanwhile, whole cerebellum also defined as reference region. The institute then applied each florbetapir scan to the corresponding MRI and calculated the mean florbetapir uptake within the cortical and reference region. Finally, ROI-based AV-45 standardized uptake value ratios (SUVR) were calculated by averaging across the three bilateral regions and dividing this average by the whole cerebellum.

### MRI scans and image processing

The structural volumetric MRI files were the original MPRAGE (T1-weighted) files downloaded from UCSF data in the ADNI dataset (https://ida.loni.usc.edu/pages/access/studyData.jsp). Morphometric analysis of brain structure was completed with FreeSurfer Version 5.1 (http://surfer.nmr.mgh.harvard.edu). Detailed methodology for regional and total volume derivation has been described in detail previously.

As reported in previous studies, although some progressive brain tissue atrophies occurred with normal aging, these atrophies were greatly accelerated in AD patients [41, 42]. These domains, which included the hippocampus, entorhinal cortex and middle temporal lobe were explored in this analysis.

### Neuropsychological assessment

The neuropsychological battery included the Mini-mental State Examination (MMSE) and the Alzheimer Disease Assessment Scale-cognitive subscale (ADAS-Cog) as tests of general cognition, and specific cognitive tests were administered to assess executive function (ADNI-EF) and memory (ADNI-MEM). The standard 11-item version of the ADAS-cog was augmented by adding 2 additional items (delayed word recall and number cancellation), and results from both the 11-item and 13-item versions were included in the ADNI dataset. ADNI-EF and ADNI-MEM composite z-scores were calculated from the ADNI neuropsychological battery of tests and have been described in detail elsewhere.

### Covariates

An extensive set of covariates for the associations of BMI with biomarkers or cognitive outcomes were evaluated at baseline in the ADNI database. They included age, sex (0=Male; 1=Female), years of education, diagnostic history of hypertension, hyperlipemia, DM2, cardiovascular disease (CVD) and depression (1 = positive diagnostic history; 0 = negative diagnostic history). Moreover, CVD status was based on a history of myocardial infarction, angina, heart failure and atrial fibrillation. APOE ε4, the best-known AD risk gene, was used as a categorical variable (number of APOE ε4 allele; 0, 1, 2).

### Statistical analysis

BMI is a recognized measure of obesity that is defined as weight in kilograms divided by the square of the height in meters. The participants in our study were categorized in accordance with the WHO classification system: normal weight (BMI = 18.5~24.9 kg/m2), overweight (BMI = 25.0~29.9 kg/m2), and obese (BMI ≥ 30.0 kg/m2). Descriptive statistics for baseline clinical characteristics, including age, sex, education, cognitive diagnosis, ApoE ε4 status and prevalent comorbidities (i.e., hypertension, hyperlipemia, DM2, depression and CVD) were initially compared among baseline BMI categories for the total samples. Clinical characteristics were compared among BMI categories using Kruskal-Wallis tests (for continuous variables) and Pearson’s chi-square tests (for categorical variables).

First, correlations of continuous BMI variables with CSF biomarkers (i.e., Aβ42, t-tau, p-tau, t-tau/Aβ42 ratio and tau/Aβ42 ratio), amyloid PET, MRI measures and cognitive test findings (i.e., ADAS-Cog11, ADAS-Cog13, MMSE, ADNI-EF and ADNI-MEM) were evaluated via Spearman correlation. Next, in the cross-sectional analyses, the effects of BMI categories on those biomarkers and cognitive test were investigated in multiple linear regression models. In these analyses, continuous response variables were tested for approximate normality and variance homogeneity, and were Box-Cox transformed when required to satisfy test criteria. Longitudinally, those biomarkers and cognitive performance over time were compared among the BMI groups by linear mixed-effects models. These models had random intercepts and slopes for time and an unstructured covariance matrix for the random effects, and included the interaction between time and BMI categories as a predictor. All the above analyses were performed both across diagnostic groups and within each diagnostic group (i.e., MCI and NC). Finally, the Kaplan–Meier method was used to estimate the relative risk of progression to AD in relation to baseline BMI categories. Cox proportional hazard model was performed to further assess the effect of late-life BMI on the risk of AD, with adjustment for age, sex, education, cognitive diagnosis, ApoE ε4 status and comorbidities.

All analyses were 2-tailed with significance set at P < 0.05 for main effects. All statistical analyses were performed with R statistical software.
